# Proposed classification for interproximal contacts of primary molars using CBCT: a pilot study

**DOI:** 10.12688/wellcomeopenres.14713.2

**Published:** 2018-09-27

**Authors:** M. Kirthiga, M. S. Muthu, G. Kayalvizhi, C. Krithika

**Affiliations:** 1 Department of Pedodontics and Preventive Dentistry, Sri Ramachandra University, Chennai, India; 2 Department of Pedodontics and Preventive Dentistry, Indira Gandhi Institute of Dental Sciences, Puducherry, India; 3 Department of Oral Medicine and Radiology, Thai Moogambigai Dental College and Hospital, Chennai, India

**Keywords:** Computed tomography, proximal contact, primary molars, cross-sectional study

## Abstract

**Background:** Interproximal contact areas in primary teeth are known to be broader, flatter, and situated more gingivally than in permanent teeth. The objective of the present study was to evaluate the different types of intact interproximal contact areas in primary teeth using cone beam computed tomography (CBCT) among children.

**Methods:** A cross-sectional study was designed with 74 contacts from 28 existing CBCT images of children aged between 3 and 14 years, obtained from the Indian Dental Education Academy, Chennai, India. The shape of the contact area was observed at three levels, the coronal, middle, and apical thirds, in three different sections of CBCT. Prevalence of the types of contact areas was expressed in the form of numbers and percentages.

**Results:** The weighted Cohen’s kappa values for inter-examiner reliability was 0.893 at baseline. Results exhibited four different types of contact areas between the primary molars, namely, O type, X type, I type, and S type, based on the shapes observed; hence, the proposed classification is referred to as OXIS. The most common pattern seen was I (66.2%), followed by X (21.6%), O (9.4%) and the least common was S (2.7%).

**Conclusion:** The three-dimensional evaluation of intact interproximal contact areas between primary molars are of four types, O,X, I and S.

## Introduction

“Contact area” is a term used to denote the proximal heights of contour of the mesial and distal surfaces of the tooth ^1^. A well-contoured, properly positioned, firm proximal contact is essential to maintain the integrity of the dental arches and the health of the supporting structures. The contact areas between primary molars are broader, flatter, and situated farther gingivally than the contact points between permanent molars ^2– 5^. Essentially, the broader proximal contact areas observed in primary teeth are likely to increase caries susceptibility, since the self-cleansing action would be reduced because of the limited movement, leading to greater plaque accumulation ^2, 6, 7^. Previous studies in this regard ^8– 13^ have focused mainly on two areas, the association of closed or open contacts with dental caries and the progression of proximal caries. Prior studies ^8, 10^ concluded that there is an increased risk of proximal caries in the posterior primary dentition if contact points are closed rather than open. Nevertheless, another study ^11^ reported that the absence of interdental spaces is weakly associated with greater caries experience in the primary dentition. In summary, results in the existing literature regarding interproximal spaces and dental caries susceptibility are controversial. Hence, a three-dimensional assessment and a classification of interproximal contacts might facilitate a complete understanding of the relationship of adjoining surfaces of teeth at different levels, namely the coronal, middle, and apical thirds. To the best of our knowledge, no previous study has investigated the three-dimensional shapes of proximal contact areas in primary teeth. Therefore, the present cross-sectional study was undertaken to evaluate the types of non-carious interproximal contact areas of primary molars in children using existing cone beam computed tomography (CBCT) images.

## Methods

### Participants

The study protocol was reviewed and approved by the Institutional Ethics Committee, Sri Ramachandra University, Chennai (IEC-NI/16/AUG/55/54). A retrospective study was designed with CBCT images of patients who presented at the Indian Dental Education Academy, Chennai, India for various dental problems between June 2011 and March 2016. After an initial screening of 74 CBCT images selected by means of convenience sampling, 28 images of good quality and with intact primary molars in at least one quadrant were selected. CBCT images of children with special health care needs or teeth with dental caries, restorations, or crowns were excluded from the study. The final sample of 28 CBCT images were from 12 girls and 16 boys aged between 3 and 14 years.

### Measurement

Two trained pediatric dentists (K.M., K.G.) participated in the data collection process. The calibration exercise was carried out by an oral and maxillofacial radiologist (K.C.) who regularly conducts hands-on workshops on CBCT assessment and interpretation. Prior to the start of the study, the program consisted of theoretical discussions followed by practical sessions on the evaluation of CBCT images. To check the diagnostic reproducibility of the inter-reliability of the investigators, 10 CBCT images were examined independently by the two aforementioned pediatric dentists. To ensure consistency in measurements, inter-examiner variability was assessed prior to and at the end of the data collection period. The weighted Cohen’s kappa value was 0.893 at baseline and 0.931 at the end of the study, which reﬂected a high degree of conformity in the examination. Any disagreement between the examiners was arbitrated by the subject expert (K.C.) to reach a consensus.

**Figure 1.**
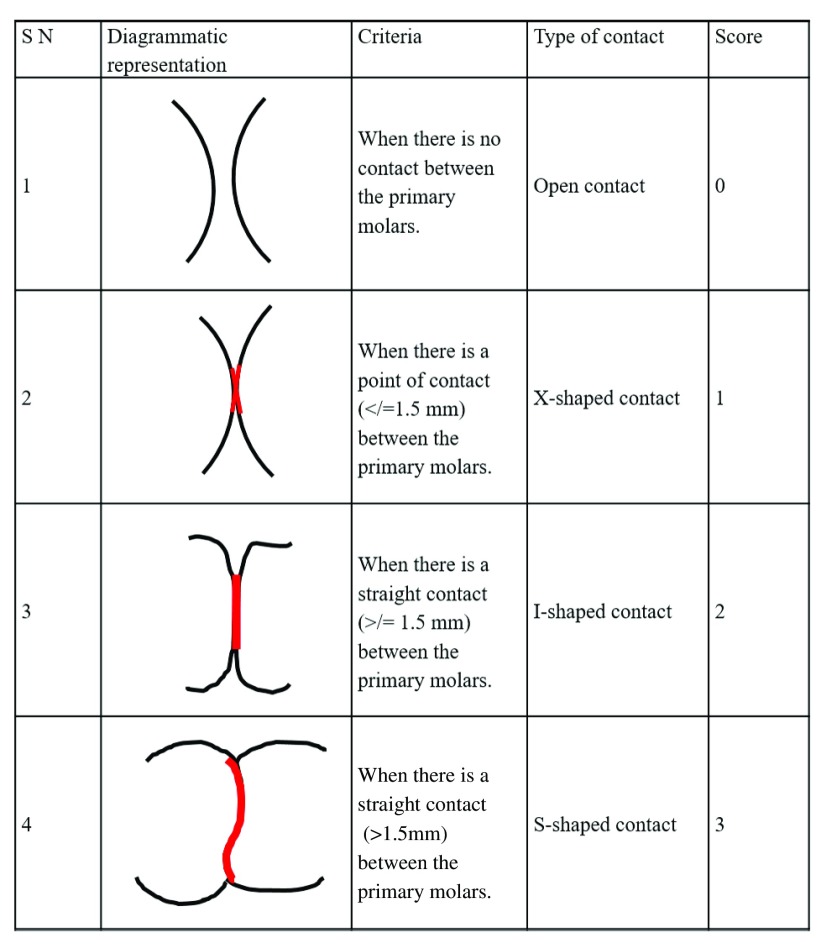
Diagrammatic representation of the type of contact according to the OXIS scoring criteria.

To ensure image standardization, all CBCT images were chosen from a single machine (Planmeca ProMax ^®^ 3D Mid) with a standard field of view = 80 mm × 80 mm; voxel size of 0.40 mm; 90 kV and 12 mA; exposure time of 12 s; and slice thickness of 0.4 mm. CBCT images were analyzed with the built-in Romexis ^®^ digital imaging software, version 3.5.2 (Planmeca, Helsinki, Finland), on a 15.6-inch Samsung LCD screen with an Intel ^®^ Core ^TM^ i3 2.4 GHz processor, and 500 GB of memory at a resolution of 1280×1024 pixels in a dark room. The observers evaluated the teeth using the Planmeca Romexis ^®^ toolbar, by carefully scrolling down through the images from the floor of the pulp chamber in all three orthogonal reconstructions (axial, coronal, and sagittal). The measurement tool was used to determine the total length of the crown of the primary second molar, measured from the tip of the mesiobuccal cusp to the cemento-enamel junction. Based on this length, the crown portion was divided into three levels: coronal, middle and apical thirds. Next, the shapes of the contact areas between the maxillary and mandibular primary molars were examined at various levels, coronal, middle, and apical, and were scored in all three sections (axial, coronal, and sagittal) according to the criteria shown in Figure 1. Depending on the maximum score among the three levels (the coronal, middle, and apical thirds), the overall score for a particular tooth was assigned. For example, if the scoring of the right maxillary contact between two primary molars was 2 at the coronal third (I shape), 1 at the middle third (X shape), and 0 at the apical third (O shape), then the overall score of this tooth would be the maximum number (that is, 2).

### Statistical analysis

Statistical analysis was performed using Microsoft Excel Version 15 (2013). Data was recorded on a custom-made data extraction sheet. ** Descriptive statistics were obtained for all independent variables. Prevalence of the types of contact areas was expressed in the form of numbers and percentages.

## Results

**Figure 2.**
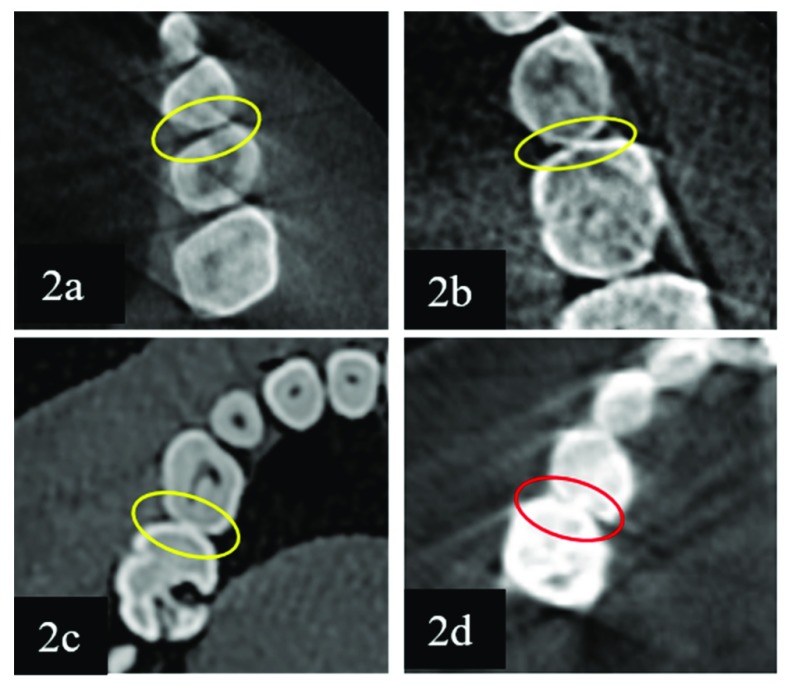
CBCT images showing different types of contact areas between primary molars. ( **a**) O type, ( **b**) X type, ( **c**) I type, and ( **d**) S type.

**Figure 3.**
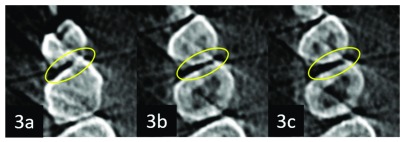
CBCT images of the interproximal contact areas of the primary molars in the maxilla. The ( **a**) coronal, ( **b**) middle, and ( **c**) apical thirds are shown, classified as X, O, and O, respectively.

**Figure 4.**
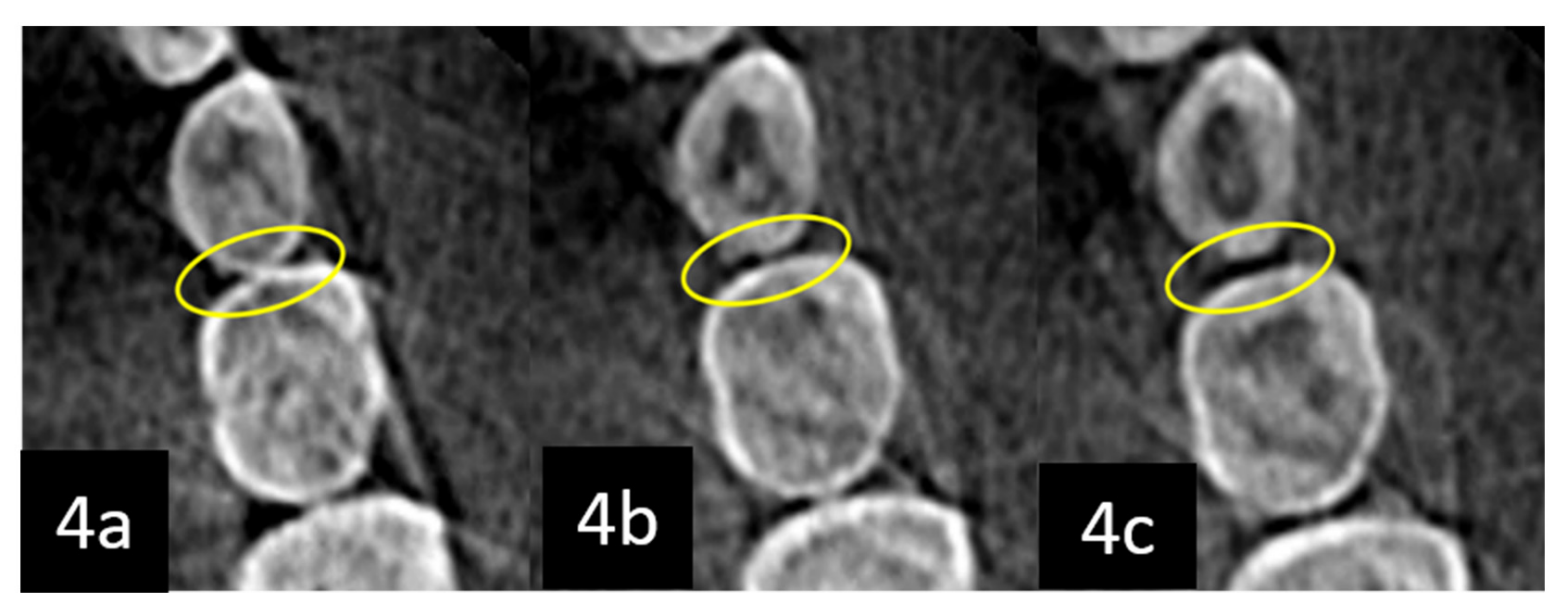
CBCT images of the interproximal contact areas of the primary molars in the mandible. The ( **a**) coronal, ( **b**) middle, and ( **c**) apical thirds are shown, classified as X, O, and O, respectively.

A total of 74 contacts from 28 CBCT images were included in the present study ^14^, of which 67 (90.5%) were of the closed type. Table 1 shows the prevalence and percentages of primary contacts according to the arch and the side. Among the different types of contacts ( Figure 2), the most common contact in the maxilla was I (67.4%) and the least was S (4.6%). In the mandible, the most and least common were I (64.5%) and S (0%), respectively. The type of contact area at the occlusal third coincided with the overall score. In addition, 65 contacts had an open contact at the middle and the apical third. The remaining nine contacts had a contact at the occlusal and middle thirds. Figure 3 and Figure 4 show CBCT images of the interproximal contact areas of the primary molars in the maxilla and mandible at the coronal, middle, and apical levels. Figure 3 shows CBCT images of the interproximal contact areas of the primary molars in the maxilla. The (a) coronal, (b) middle, and (c) apical thirds are shown, classified as X, O, and O, respectively. Figure 4 shows CBCT images of the interproximal contact areas of the primary molars in the mandible. The (a) coronal, (b) middle, and (c) apical thirds are shown, classified as X, O, and O, respectively.

**Table 1.** Prevalence and percentages of primary contacts according to the arch and side.

Maxilla (n = 43)					Mandible (n = 31)			
Type of contact	Right side (n = 21)	Left side (n = 22)	%	% closed contacts	Right side (n = 15)	Left side (n = 16)	%	% closed contacts
Open (0)	3	3	13.9	0	1	0	3	0
X (1)	1	5	13.9	16.2	2	8	32.2	33.3
I (2)	15	14	67.4	78.3	12	8	64.5	66.6
S (3)	2	0	4.6	5.4	0	0	0	0

Right side vs left side of maxilla: χ ^2^ = 26.48, *P* = 0.001 (< 0.05). Right side vs left side of mandible: χ ^2^ = 4.33, *P* = 0.228.

## Discussion

The present study used existing multi-planar CBCT scans for the preliminary classification of the contact areas of primary molars in a retrospective manner and is proposed as the OXIS classification. However the prescription of CBCTs is not recommended for the study of contact areas in children. As radiation exposure in children and young people (adolescents) is associated with greater risk of stochastic effects, appropriate use in paediatric dentistry is essential. The most frequent uses of CBCT in children are for impacted supernumerary teeth, disorders in tooth eruption, for temporomandibular joint (TMJ) investigation, localisation of unerupted teeth and the identification of resorption in relation to unerupted teeth ^15^. The types of proximal contacts between primary molars can be visualized clearly in axial, coronal, and sagittal sections at three different levels, namely the coronal, middle, and apical thirds. The interproximal contacts were named according to the shape in which they were observed. The criteria for scoring at each level for each contact area were as described in the Methods ( Figure 1). The scoring according to the numbers 0 to 3 to increase in the surface area of contact between the primary molars. This classification for non-carious interproximal contacts of primary molars based on CBCT observations is proposed as the OXIS classification.

Earlier studies ^8, 10, 11^ used different criteria to determine the nature of the contacts or the spacing between the primary molars. The closed/open nature of the contact point was assessed by passing a dental floss through the interproximal contact point ^8^. Of these previous studies, two ^10, 11^ evaluated tooth spacing in primary teeth on a space-to-space basis based on the following criteria: (i) spacing present >1 mm; (ii) spacing present but <1 mm; (iii) spacing not present, teeth in contact; or (iv) spacing not present, teeth overlapping. These criteria were not used in the present study, since they classified only the open/closed nature and not the specific type of contact. In the present study, 90.5% of the contacts were closed, which was comparable with results reported in previous studies ^8, 10^, where 84% and 90% were observed. The number of closed contacts was greater in the maxilla than in the mandible, which again was in accordance with results from a former study ^10^. Previous studies in this area evaluated the relationship between the closed/open nature of contact points or spacing between teeth and interproximal caries ^8, 10, 11^. Their results are in agreement with the concept that the absence of interdental spaces in the primary dentition may alter plaque accumulation and cause difficulty in mechanical cleansing. This could sequentially contribute to increased caries susceptibility. Nevertheless, the specific shape of the contact area has, to our knowledge, not been previously studied. An understanding of the proximal contact area in a three-dimensional manner has increased the need for this to be considered a potential risk factor for caries risk assessment. Another clinical implication is that the change in the type of contact area (open or closed) may also influence the cavity preparation in primary teeth especially in class II preparations.

Two interesting observations were made in the present study. First, in all the contacts studied, the type of contact area at the occlusal third coincided with the overall score, indicating that the contact area existed only at the occlusal third of the tooth surface. Hence, it may be sufficient to observe the occlusal third alone, rather than the three levels. The second observation was that, of the 74 contacts, 65 had an open contact at the middle third, and all the contacts were open at the apical third. This also raises questions regarding existing knowledge ^2– 5^, which states that contact areas between primary molars are broader, flatter, and situated farther gingivally. The small sample size, however, could be seen as a limitation of the present study.

## Conclusion

In conclusion, the contact areas vary as four different types, namely Open, X-shaped, I-shaped, and S-shaped; hence, we propose the OXIS classification of primary molars. Further, the three-dimensional evaluation of intact interproximal contact areas between primary molars indicated that the contact area is predominantly present at the occlusal level.

## Data availability

Raw data associated with this study, including the images used to assess contact areas and the measurement of contact areas themselves, are available on OSF: https://doi.org/10.17605/OSF.IO/N2FCE ^14^. Data are available under the terms of the Creative Commons Zero “No rights reserved“ data waiver (CC0 1.0 Public domain dedication).
