# Supplementary material for: Proposed classification for interproximal contacts of primary molars using CBCT: a pilot study
Source: Wellcome Open Res. 2018 Sep 27;3:98. Originally published 2018 Aug 9. [Version 2] doi: 10.12688/wellcomeopenres.14713.2 (PMC6171554; doi:10.12688/wellcomeopenres.14713.2)
Supplement: Peer review history

# Peer review: Referee response for version 2

**DOI:** 10.21956/wellcomeopenres.16184.r34003

**Published:** 2018-09-27

**Authors:** Sivakumar Nuvvula

**Affiliations:** 1 Department of Pedodontics and Preventive Dentistry, Narayana Dental College and Hospital, Nellore, Andhra Pradesh, India

I have read this submission. I believe that I have an appropriate level of expertise to confirm that it is of an acceptable scientific standard.

# Peer review: Referee response for version 1

**DOI:** 10.21956/wellcomeopenres.16025.r33817

**Published:** 2018-09-20

**Authors:** Manikandan Ekambaram

**Affiliations:** 1 Paediatric Dentistry, Faculty of Dentistry, University of Otago, Dunedin, New Zealand

General comments:

 It is an interesting study and has clinical relevance. The authors of the study have used CBCT images for examination of the interproximal contact surfaces though this is not a routine practice. However, the CBCT images were previously taken for purposes not related to the present study and therefore, this would not raise any concerns of exposing children to ionizing radiation for the study objective.

 Specific comments:

 Abstract:

 Background: “Contact areas in primary teeth…..teeth” should read as “Interproximal contact areas in primary teeth…..teeth”. Similarly, “The objective…….of intact contact areas in primary teeth…..among children” should read as ““The objective…….of intact interproximal contact areas in primary teeth…..among children”. Methods: Please delete “obtained from the Indian Dental Education Academy, Chennai, India”. The sentence “The weighted Cohen’s kappa…..at baseline” should be moved to the results part of the abstract.

 Introduction:

 Paragraph 1, line number 5 and 6, please delete the phrase “Several pediatric dentistry textbooks have suggested that”

 Methods:

 Participants:

 The last few lines could be rewritten as follows:

 The selected CBCT images did not consist images from children with special health care needs or teeth with dental caries, restorations, or crowns. The final sample of 28 CBCT images were from 12 girls and 16 boys aged between 3 and 14 years.

 Measurement:

 The sentence “There were two trained…..data collection process” should read as “Two trained pediatric dentists (K.M., K.G.) participated in the data collection process”.

 Paragraph 3, in the last few sentences, for the overall scoring criteria, score 3 for “I” shape, score 2 for “X” shape and score 1 for “O” shape does not match with the scoring criteria mentioned in figure 1.

 Table 1

 The heading of column 1, should be changed to “Type of contact(score)”.

I have read this submission. I believe that I have an appropriate level of expertise to confirm that it is of an acceptable scientific standard.

# Peer review: Referee response for version 1

**DOI:** 10.21956/wellcomeopenres.16025.r33678

**Published:** 2018-08-23

**Authors:** Sivakumar Nuvvula

**Affiliations:** 1 Department of Pedodontics and Preventive Dentistry, Narayana Dental College and Hospital, Nellore, Andhra Pradesh, India

Good study exploring the areas that were never touched before in Pediatric dentistry,

 However, there are certain language and typo errors in the abstract as well as the article text.

 For example: The word " **patients**" in Methods section of abstract can be changed to " **children**". Scoring of the type of contact in **Methods** section ( **Figure 1**) is mentioned as **0 to 3**, however, in the **Discussion** part, it is mentioned as **1 to 4**. It should read, 0 to 3 rather than 1 to 4. Furthermore, the second line of the Discussion says, " **However the prescription of CBCTs is not recommended for the study of contact areas in children**." Authors can add detail on why CBCT is not recommended as a routine investigation as well as the special conditions in which it is indicated.

 I have marked the language corrections on the PDF file available here.

I have read this submission. I believe that I have an appropriate level of expertise to confirm that it is of an acceptable scientific standard.
